# Achieving Textbook Outcomes in Colorectal Cancer Surgery Is Associated with Improved Long-Term Survival: Results of the Multicenter Prospective Cohort Study

**DOI:** 10.3390/jcm13051304

**Published:** 2024-02-25

**Authors:** Marius Kryzauskas, Augustinas Bausys, Vilius Abeciunas, Austeja Elzbieta Degutyte, Klaudija Bickaite, Rimantas Bausys, Tomas Poskus

**Affiliations:** 1 Clinic of Gastroenterology, Nephrourology, and Surgery, Institute of Clinical Medicine, Faculty of Medicine, Vilnius University, 03101 Vilnius, Lithuania; tomas.poskus@santa.lt; 2 Department of Abdominal Surgery and Oncology, National Cancer Institute, 08660 Vilnius, Lithuania; rimantas.bausys@nvi.lt; 3 Faculty of Medicine, Vilnius University, 03101 Vilnius, Lithuania; vilius.abeciunas@gmail.com (V.A.); austeja.elzbieta.degutyte@gmail.com (A.E.D.); klaudija.bickaite@santa.lt (K.B.)

**Keywords:** colorectal cancer, textbook outcome, cancer survival, long-term outcomes

## Abstract

**Background**: The outcomes of patients with colorectal cancer greatly depend on the quality of their surgical care. However, relying solely on a single quality indicator does not adequately capture the multifaceted nature of modern perioperative care. A new tool—“Textbook Outcome” (TO)—has been suggested to provide a comprehensive evaluation of surgical quality. This study aims to examine how TO affects the long-term outcomes of colorectal cancer patients who are scheduled for surgery. **Methods**: The data of all patients undergoing elective colorectal cancer resection with primary anastomosis at two major cancer treatment centers in Lithuania—Vilnius University Hospital Santaros Klinikos and National Cancer Institute—between 2014 and 2018 were entered into the prospectively maintained database. The study defined TO as a composite quality indicator that incorporated seven parameters: R0 resection, retrieval of ≥12 lymph nodes, absence of postoperative complications during the intrahospital period, hospital stay duration of fewer than 14 days, no readmission within 90 days after surgery, no reinterventions within 30 days after surgery, and no 30-day mortality. Long-term outcomes between patients who achieved TO and those who did not were compared. Factors associated with failure to achieve TO were identified. **Results**: Of the 1524 patients included in the study, TO was achieved by 795 (52.2%). Patients with a higher ASA score (III-IV) were identified to have higher odds of failure to achieve TO (OR 1.497, 95% CI 1.203–1.863), while those who underwent minimally invasive surgery had lower odds for similar failure (OR 0.570, 95% CI 0.460–0.706). TO resulted in improved 5-year overall—(80.2% vs. 65.5%, *p* = 0.001) and disease-free survival (76.6% vs. 62.6%; *p* = 0.001) rates. **Conclusions**: Elective colorectal resections result in successful TO for 52.5% of patients. The likelihood of failure to achieve TO is increased in patients with a high ASA score, while minimally invasive surgery is associated with higher TO rates. Patients who fail to achieve successful surgical outcomes experience reduced long-term outcomes.

## 1. Introduction

Colorectal cancer (CRC) is the third most prevalent cancer worldwide, with over 1.9 million new cases annually [1]. The survival of CRC patients depends on multiple factors, including the stage of disease at diagnosis, patient characteristics, tumor biology, and treatment differences [2,3]. Surgery is the cornerstone of treatment, but radiation and chemotherapy schemes are typically incorporated before or after the surgery [4]. Advancements in multimodal colorectal cancer treatments have led to improved outcomes, but significant survival differences still exist [3,4,5]. While various indicators have been used to compare hospitals, treatment protocols, and surgeons, they do not fully reflect the complexity of perioperative care [6,7]. Patients have also expressed a preference for concise, summarizing measures while evaluating individual surgeons and hospitals [8].To address this, a composite measure called “Textbook Outcome” (TO) has been developed, which encompasses all desirable outcomes and requires patients to meet all critical quality-of-care parameters for the procedure [9,10]. TO is an efficient predictor of survival and is also considered an indicator of the quality of care hospitals provide. A 2013 Dutch study found that TO represents the proportion of patients with perfect hospitalization and provides a comprehensive summary of hospital performance, making it a meaningful parameter for patients, providers, insurance companies, and the healthcare inspectorate [10]. Several articles have emerged analyzing the impact of TO on long-term outcomes in colon cancer patients [3,11]. Nonetheless, a gap still remains in the available data regarding the impact of TO on long-term outcomes within the broader subgroup of colorectal cancer patients.

Therefore, this study aims to investigate the impact of TO on the long-term survival of patients with colorectal cancer using this novel composite quality indicator.

## 2. Materials and Methods

### 2.1. Ethical Statement

The study was conducted in accordance with the Declaration of Helsinki and was approved by the Vilnius Regional Research Ethics Committee (26 March 2019; No. 2019/3-116-608), which granted a waiver for informed consent.

### 2.2. Study Setting and Patients

**Figure 1:**
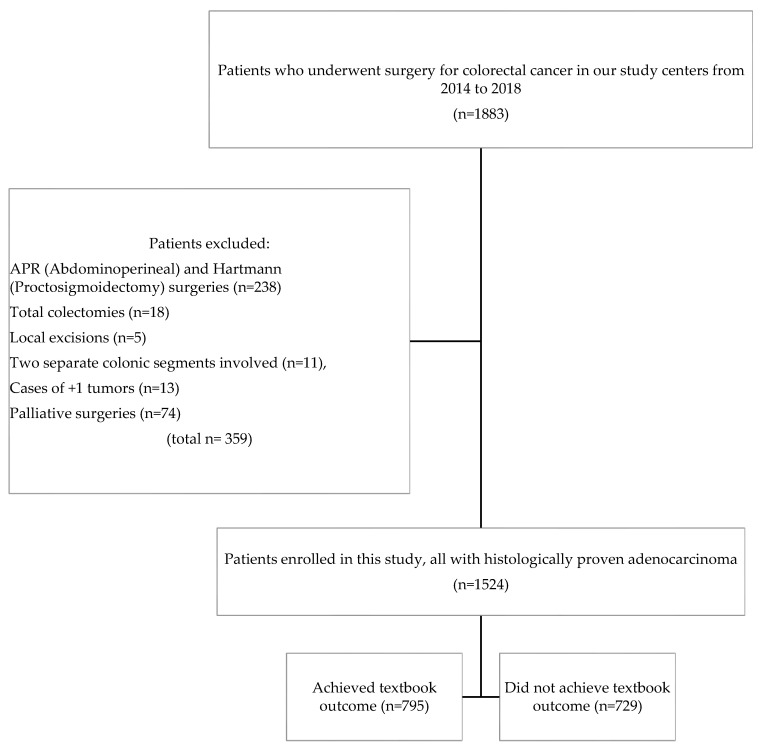
Flowchart of the patient selection process.

The data of all patients undergoing elective curative colorectal cancer resection at two major cancer treatment centers in Lithuania—Vilnius University Hospital Santaros Klinikos and National Cancer Institute—between 2014 and 2018 (Figure 1) were entered into a prospectively maintained database. Exclusion criteria were any of the following: abdominoperineal (APR), Hartmann surgeries, total colectomies, multiple tumors, separate colonic segment involvement, and palliative surgeries. The necessary data for this study, including age, gender, American Society of Anesthesiologists (ASA) score, tumor stage and location, type of surgery, R0 resection rate, number of retrieved lymph nodes, postoperative complications, classified according to the Clavien–Dindo classification [12], duration of hospital stay, history of readmission after initial discharge, were obtained from these databases. The tumor stage was coded using the TNM system described in the Union Internationale Contre le Cancer/American Joint Committee on Cancer 8th edition. To evaluate long-term outcomes, 5-year disease-free survival (DFS) and overall survival (OS) rates were obtained from the Lithuanian National Cancer Registry, which is prospectively maintained and has a registration rate of over 98%.

### 2.3. Textbook Outcome Definition

The study defined Textbook Outcome as a composite quality indicator incorporating seven parameters related to the oncologic quality of surgical resection and the postoperative course. These parameters were R0 resection, retrieval of ≥12 lymph nodes, absence of postoperative complications during the intrahospital period, hospital stay duration of fewer than 14 days, no readmission within 90 days after surgery, no reinterventions within 30 days after surgery, and no 30-day mortality. To achieve Textbook Outcome, all seven parameters had to be met.

### 2.4. Study Outcomes

The study’s primary outcome was overall survival, defined as the time from surgery to death. The last day of the follow-up was the 1st of June, 2021. The secondary outcome included disease-free survival, defined as the time from surgery to the progression of the disease or death from any cause. Factors associated with Textbook Outcomes were also analyzed. 

### 2.5. Statistical Analysis 

All statistical analyses were performed by the statistical package SPSS 25.0 (IBM SPSS, Chicago, IL, USA). Patients were grouped into those who had achieved textbook outcomes and those who did not achieve textbook outcomes. Data were checked for normality and were expressed as proportions with percentages. Overall- and disease-free survival was estimated by the Kaplan–Meier method, and the log-rank test compared curves. Univariate analysis was performed to reveal factors associated with Textbook Outcome, and variables with significance were included in subsequent multivariable logistic regression. 

## 3. Results

### 3.1. Study Patients 

A total of 1524 patients with colorectal adenocarcinoma who underwent surgery were identified. Of these, 795 (52.16%) achieved a textbook outcome. All demographic and operative characteristics are presented in Table 1. Younger age (<75 years) (54.4% vs. 45.6%, *p* < 0.001), lower ASA score (I–II) (56.9% vs. 43.1% *p* < 0.001), and minimally invasive surgical approach (61.5% vs. 38.5%, *p* < 0.001) were associated with TO. 

### 3.2. Contributors of Individual Parameters to Textbook Outcome

**Figure 2:**
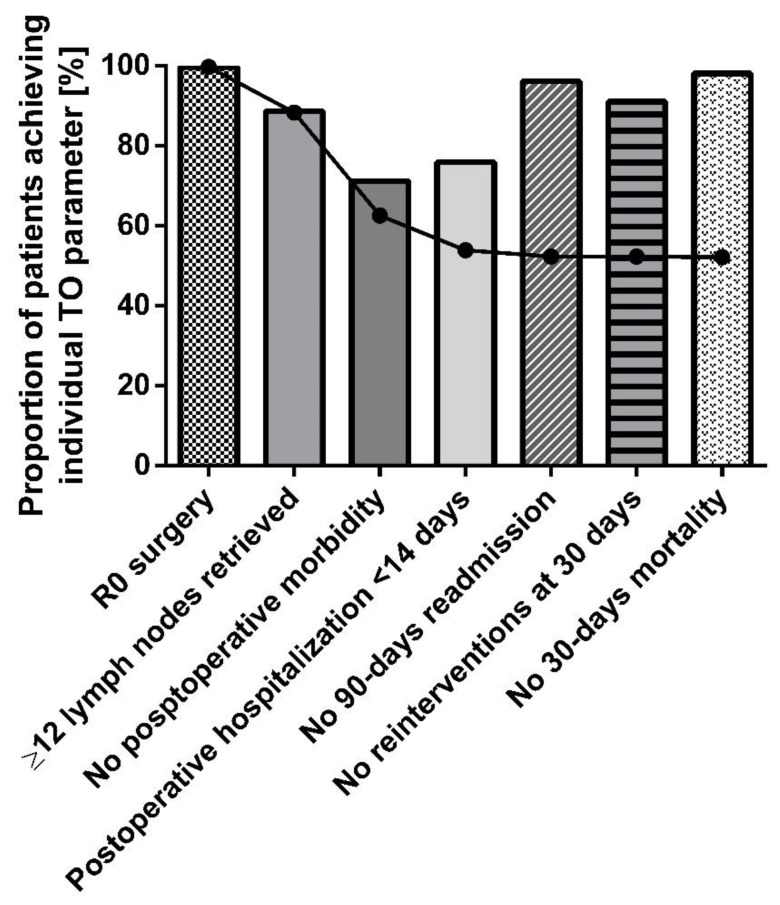
Individual parameters in TO.

The bar chart below (Figure 2) shows cumulative percentages of the individual TO parameters. Postoperative complications had the most detrimental impact on the textbook outcome of all individual parameters since it was met by the least number of patients (a decrease of 28.5%). The TO parameters most easily met were tumor-free margins, no readmission in the next 90 days, and no 30-day mortality, all of which were met by almost all patients.

### 3.3. Association of Textbook Outcome and 5-Year DFS and OS Rates

**Figure 3:**
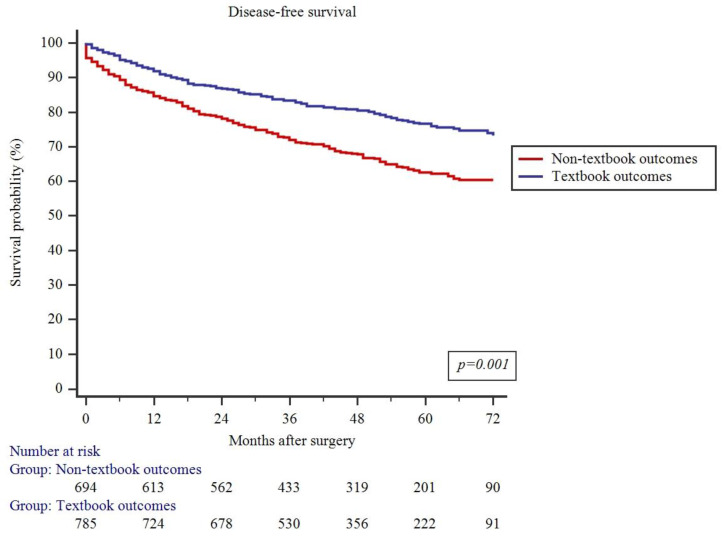
Impact of textbook outcomes on disease-free survival.

**Figure 4:**
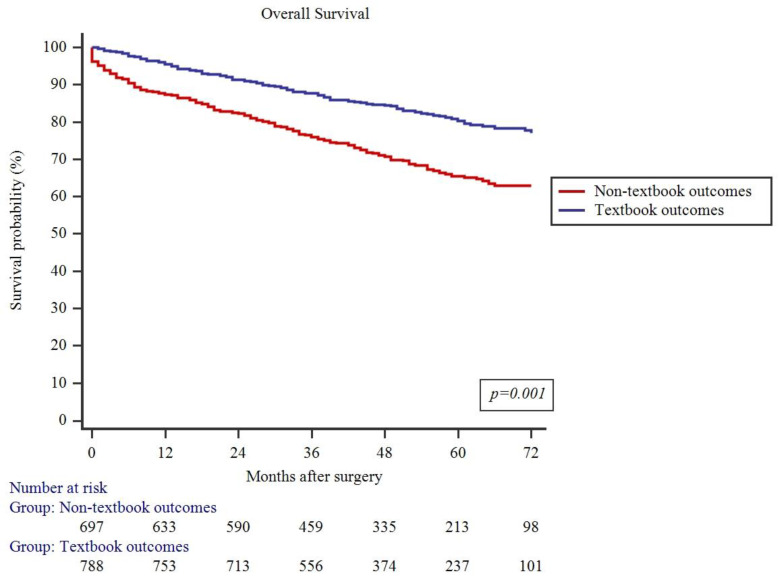
Impact of textbook outcomes on overall survival.

As summarized in Figure 3, failure to achieve TO resulted in decreased 5-year DFS (62.6% vs. 76.6%, *p* = 0.001). Similarly, 5-year OS was also lower in patients who failed to achieve TO (65.5% vs. 80.2%, *p* = 0.001) (Figure 4).

### 3.4. Factors Associated with Textbook Outcome 

Multivariable analysis identified higher (III-IV) ASA scores to be associated with lower odds of achieving TO (OR 1.497, 95% CI 1.203–1.863, *p* < 0.001). Patients who underwent minimally invasive (MI) surgery had lower odds of failing to achieve TO (OR 0.570, 95% CI 0.460–0.706, *p* < 0.001) (Table 2).

### 3.5. Association of Textbook Outcome and Surgeon Volume

**Figure 5:**
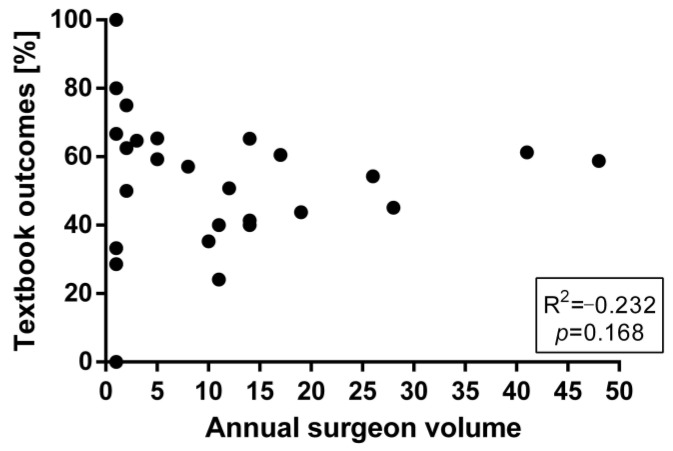
Impact of annual surgeons’ volume on textbook outcomes.

Individual annual surgeon’s volume did not correlate with the proportion of patients achieving TO (R^2^ = −0.232, *p* = 0.168) (Figure 5).

## 4. Discussion

This large cohort study showed that 52.5% of patients achieve TO after elective surgery for colorectal cancer. Patients with higher ASA scores (III-IV) were identified to have higher odds of failure to achieve TO (OR 1.497, 95% CI 1.203–1.863), while those who underwent minimally invasive surgery had lower odds for similar failure (OR 0.570, 95% CI 0.460–0.706). TO is associated with improved long-term outcomes after elective colorectal cancer surgery.

While the concept of textbook outcome has been recognized for more than a decade, the individual parameters comprising TO are still varying [10]. Originally introduced for colorectal cancer surgery by Kolfschoten, TO was assessed by six distinct outcome measures: hospital survival, R0 resection, absence of ostomy, no reintervention, no adverse outcomes, and a hospital stay of 14 days or less [10]. While the concept of TO aims to establish a standardized benchmark for evaluating the quality of surgical management, variation of individual parameters between authors still exists. For instance, an article published by Maeda et al. modeled TO based only on five different parameters: surgery within 6 weeks, radical resection, LN yield ≥12, absence of stoma, and no adverse outcome [3]. This diversity in parameter selection highlights the ongoing efforts within the medical community to refine the concept of textbook outcomes. Additionally, Azevedo et al.’s recent research on robotic colorectal cancer resections formulated TO, based on no conversions to open and no complications with a Clavien–Dindo score of ≥3, LOS of ≤14, radical resection, no 30-day readmission, and no 30-day mortality [13]. The decision to include only complications with a CD score of ≥3 contrasts with the approach taken in the majority of publications and our data, where all complications were considered in the assessment of TO. Consequently, the percentage of successfully achieved TO is notably higher, observed in 77.4% of the patients [13]. Certain authors emphasize the lack of a stoma post-surgery to be a crucial component of TO [14]. However, in high-risk low anastomosis cases, a preventive ileostomy is typically instituted as a standard preventive measure against anastomotic leakage or to minimize potential damage from such leakage. Thus, abdominoperineal resection inclusion in the study would greatly reduce the percentage of patients able to attain textbook outcome. Recently, a new term has been introduced—modified textbook outcome (mTO)—where authors divert from the standard textbook outcome parameters [15,16]. Length of hospital stay is commonly excluded from textbook outcome definitions due to differences in protocols; for example, certain hospitals conduct cancer staging within the in-patient setting, leading to an extension of the overall duration of hospitalization [3]. This poses a challenge for comparing different trials as the LOS ranks among the least met in the existing literature [13]. It is imperative to underscore the necessity for standardizing the definition across different categories, given that a textbook outcome formulated with a more lenient set of five individual parameters may be achieved more frequently than a more stringent formulation composed of seven parameters. We believe the seven-parameter model proposed in this study is the most comprehensive and the best reflector of surgical quality.

In modern healthcare systems, achieving TO has become increasingly important. Recent research has highlighted the crucial role of TO in ensuring the safer delivery of healthcare services [17,18,19]. TO is defined as a combination of different outcome measures that can provide greater value compared to individual parameters when conducting clinical audits of surgical treatments, according to Fukuoka A and colleagues [20]. Our study showed that only 52.5% of patients undergoing elective surgery for colorectal cancer achieved TO. Although univariate analysis showed a lower proportion of elderly patients (≥75) achieving TO, this factor was not significant in multivariable analysis. These results contradict Fukuoka and colleagues’ previous findings, who reported that patients older than 85 were more prone to postoperative complications, particularly pneumonia and thromboembolism [20]. However, the age threshold used in our study was significantly different, so the findings may not be entirely consistent. Nonetheless, special attention should be paid to delivering the highest quality care to elderly patients. Warps et al. reported gender-specific differences in achieving TO in rectal cancer patients, with males at a higher risk of failure [21]. Similarly, an increased risk of failure was reported for males undergoing distal pancreatectomy [22]. However, our study did not identify gender-specific differences in TO rates after colorectal cancer resections. This suggests that risk factors, such as complex surgery in the male pelvis, can be mitigated by performing surgery in high-volume centers. Despite these differences, our study and previous research consistently show that postoperative complications are the main driving factor for failure to achieve TO [21,22]. Therefore, it is unsurprising that our multivariable analysis showed increased odds of failure in patients with a high ASA score. A high ASA score is a well-described risk factor for postoperative complications and has been associated with TO failure in patients with metastatic colorectal cancer after liver resection [23]. A minimally invasive approach may increase the probability of achieving TO in patients undergoing proctectomy [24]. Our study identified that a similar approach could benefit all patients with colorectal cancer. Therefore, maximizing the percentage of surgeries performed using a minimally invasive approach is essential.

The success of surgical procedures is often attributed to the skill of individual surgeons [25]. Birkmeter et al. underscored the relationship between surgeon and hospital volume and the subsequent decrease in operative mortality more than two decades ago [26]. This relation is one of the main drivers of complex cancer surgery centralization in several countries. Consolidation of complex cases promises to improve patient care by collating expertise, and cutting-edge equipment within centers of excellence [27]. However, the association between long-term outcomes of cancer patients and surgical volume is conflicting to this day. The impact of surgical case volume on long-term survival has been extensively analyzed in hepatopancreatic and gastric cancer surgery [28,29,30,31]. However, the relationship between caseload and textbook outcome is still conflicting. Mehta et al. reported greater odds of achieving textbook outcomes after hepatopancreatic surgery for cancer at major teaching hospitals, highlighting that the procedural volume was the main mediator [29]. Levy et al. emphasized that increased case volume may influence specific aspects of quality of care. However, it is noteworthy that neither the volume of surgeons nor hospitals showed a statistically significant association with the textbook outcome for patients undergoing gastrectomy [31]. Several articles have analyzed the association of textbook outcome and surgical case volume with long-term survival [28,30]. Kalagara et al. reported that improved long-term survival following pancreatic resection was associated with TO rather than high hospital volume [28]. This contrasts with the findings of Khalil et al., who reported that prolonged survival following hepatocellular carcinoma resection was largely associated with hospital case volume rather than TO [30]. The available research on the relationship between surgical caseload and textbook outcomes in colorectal cancer surgery is still sparse. Our study did not discern any statistically significant difference in surgical outcomes between low- and high-volume surgeons, thus casting doubt on this hypothesis. Instead, it appears that the volume of the hospital where the surgery takes place is a more critical factor in achieving positive outcomes for colorectal cancer patients. For instance, low-volume units (with fewer than 50 cases per year) were found to have a higher rate of failures in a previous study by Sweigert PJ et al. [32]. Failure to achieve TO can have a detrimental impact on long-term patient survival, as has been shown in cases of colectomy [3,11], hepatic metastases [33] and primary liver cancer [34] surgery, esophagectomy [35,36,37], and pancreatectomies [38]. Our study confirms that failure to achieve TO after colorectal cancer surgery is associated not only with reduced disease-free survival but also with impaired overall survival. Moreover, the rate of excellent surgical outcomes, as measured by the TO parameter, is increasingly used as an indicator of the quality of care hospitals provide. It is suggested that this parameter should not only be used to evaluate individual hospitals but also national performance. Additionally, it provides an opportunity to provide benchmarked feedback to surgeons about the effectiveness of a hospital and its care quality [39]. 

The strengths of this study include a large sample size and a multicenter approach. However, it also has some limitations that should be considered. The study’s retrospective design introduces a risk of selection bias, potentially impacting the identification of factors that may affect TO. Additionally, our databases did not include some relevant variables, such as BMI, type of neoadjuvant treatment, and comorbidities, as assessed by the Charlson comorbidity index. Moreover, our analysis did not include patients undergoing surgery without primary anastomosis formation. The homogeneity of our cohort, with a predominantly Caucasian ethnicity, prevented us from evaluating the potential impact of race on the TO. Despite these limitations, our study demonstrates that a significant proportion (47.5%) of patients with colorectal cancer fail to achieve successful surgical outcomes following elective surgery. Such failure is associated with a higher ASA score, but patients who undergo minimally invasive surgery are at lower risk. Furthermore, we observed that the failure to achieve successful surgical outcomes results in decreased long-term outcomes.

## 5. Conclusions

Elective colorectal resections result in successful TO for 52.5% of patients. The likelihood of failure to achieve TO is increased in patients with a high ASA score, while minimally invasive surgery is associated with higher TO rates. Patients who fail to achieve successful surgical outcomes experience reduced long-term outcomes.

## Figures and Tables

**Table 1:** Baseline characteristics of the study population stratified for textbook outcome.

		Textbook Outcome (n = 795)	No Textbook Outcome (n = 729)	*p*-Value
Age	<75	640 (54.4%)	536 (45.6%)	0.001
	≥75	155 (44.5%)	193 (55.5%)	
Gender	Female	401 (52.6%)	361 (47.4%)	0.758
	Male	394 (51.7%)	368 (48.3%)	
ASA	I–II	526 (56.9%)	399 (43.1%)	<0.001
	III–IV	269 (44.9%)	330 (55.1%)	
Tumor location	Cecum	54 (45.4%)	65 (54.6%)	0.356
	Ascending colon	128 (53.3%)	112 (46.7%)	
	Hepatic flexure	32 (55.2%)	26 (44.8%)	
	Transverse colon	25 (45.5%)	30 (54.5%)	
	Splenic flexure	32 (60.4%)	21 (39.6%)	
	Descending colon	39 (57.4%)	29 (42.6%)	
	Rectosigmoid	71 (56.8%)	54 (43.2%)	
	Sigmoid colon	165 (54.5%)	138 (45.5%)	
	Rectum	249 (49.5%)	254 (50.5%)	
Colon or rectal cancer	Colon	546 (53.4%)	476 (46.6%)	0.173
	Rectum	249 (49.6%)	253 (50.4%)	
T stage	T1–T2	196 (50.3%)	194 (49.7%)	0.411
	T3–T4	593 (52.7%)	532 (47.3%)	
N stage	N0	466 (51.7%)	436 (48.3%)	0.639
	N+	329 (52.9%)	293 (47.1%)	
M stage	0	745 (52.6%)	671 (47.4%)	0.231
	1	50 (46.3%)	58 (53.7%)	
TNM Stage	1	157 (49.5%)	160 (50.5%)	0.308
	2	292 (53.0%)	259 (47.0%)	
	3	297 (54.1%)	252 (45.9%)	
	4	49 (45.8%)	58 (53.2%)	
Surgical approach	Open	447 (46.7%)	511 (53.3%)	<0.001
	MI	348 (61.5%)	218 (38.5%)	
Type of surgery	Right hemicolectomy	227 (51.0%)	218 (49.0%)	0.204
	Transverse colectomy	13 (46.4%)	15 (53.6%)	
	Left hemicolectomy	93 (55.0%)	76 (45.0%)	
	Sigmoidectomy	149 (58.0%)	108 (42.0%)	
	Rectosigmoidectomy	26 (42.6%)	35 (57.4%)	
	Rectal resection	287 (50.9%)	277 (49.1%)	

Data are n (%). ASA, American Society of Anesthesiologists classification score; MI, minimally invasive.

**Table 2:** Multivariate analyses to determine factors predictive of textbook outcome.

	*Risk Factor*	*Odds Ratio*	*95% C.I.*	*p-Value*
Age	>75 years	1.207	0.935–1.559	0.149
ASA	III–IV	1.497	1.203–1.863	<0.001
Surgical approach	Minimally invasive	0.570	0.460–0.706	<0.001

ASA: American Society of Anesthesiologists’ classification score.

## Data Availability

The datasets generated during and/or analyzed during the current study are available from the corresponding author upon reasonable request.
